# Heroin-induced osteoporosis presented with bilateral femoral neck insufficiency fractures in a male adult: a case report

**DOI:** 10.1186/s12891-023-06377-y

**Published:** 2023-04-14

**Authors:** Yu-Jen Shih, Wei-Ning Chang, Shan-Wei Yang

**Affiliations:** 1 Department of Orthopedics, Kaohsiung Veterans General Hospital, 386 Ta-Chung 1St Road, Kaohsiung, Taiwan 81346; 2 School of Nursing, Fooyin University, Kaohsiung, Taiwan

**Keywords:** Osteoporosis, Femoral neck fracture, Opioid, Case report

## Abstract

**Background:**

Osteoporosis has been associated with several disorders; however, there have been only a limited number of reports on heroin-induced osteoporosis. We report a rare case presented with bilateral femoral neck insufficiency fractures without trauma history, caused by heroin-induced osteoporosis. We collect sufficient clinical data and further shed light on the potential mechanism of how heroin affects bone formation and decreases bone density.

**Case presentation:**

A 55-year-old male patient with normal body mass index (BMI) suffered from bilateral hips pain gradually without trauma history. He had intravenous heroin addiction for more than 30 years. Radiography revealed bilateral femoral neck insufficiency fractures. Laboratory tests showed elevated alkaline phosphatase levels (365 U/L) and decreased inorganic phosphate (1.7 mg/dL), calcium (8.3 mg/dL), 25-(OH)D3 (20.3 ng/ml) and testosterone levels (2.12 ng/ml). Magnetic resonance imaging (MRI) revealed increased signals on STIR images over the sacral ala and bilateral proximal femur, and multiple band-like lesions at the vertebrae of the thoracic and lumbar spine. Bone densitometry revealed osteoporosis with a T score of minus 4.0. The screen for urine morphine was positive (> 1000 ng/ml). Through assessment of the patient, the diagnosis was insufficiency fractures of bilateral femoral neck caused by opioid-induced osteoporosis. After hemiarthroplasty, regular medication with vitamin D3 and calcium, and detoxification treatment, and the patient recovered well after 6 months of follow-up.

**Conclusion:**

The aim of this report is to highlight the laboratory and radiology findings in a case of osteoporosis caused by opioid addiction and discuss the potential pathway by which osteoporosis is induced by opioids. When an unusual osteoporosis presents with insufficiency fractures, heroin-induced osteoporosis should be considered.

## Background

Osteoporosis is a skeletal disorder characterized by reduced bone mass and impaired bone microarchitecture which further results in debilitated bone strength and increased possibility of fracture risk [1]. Although most patients are asymptomatic initially, almost all major osteoporotic fractures have shown increased mortality risk [2]. Osteoporosis is associated with several risk factors, mainly age and estrogen deficiency caused by menopause [3]. A previous study mentioned that chronic use of opioid drugs could have a negative impact on bone metabolism and thus reduce trabecular bone mass [4].

In patients with opioid addiction complicated with osteoporosis, the diagnostic method and therapeutic plan rely on clinical manifestations, laboratory examinations and radiology images. Herein, we report a rare case of a male with bilateral femoral neck insufficiency fracture caused by opioid-induced osteoporosis. In this case, we collect sufficient clinical data and further shed light on the potential mechanism of how heroin affects bone formation and decreases bone density.

## Case presentation

A 55-year-old male presented with bilateral hip pain and claudication without trauma. He was referred to our orthopaedics department from the medical department due to severe osteoporosis and bilateral femoral neck insufficiency fractures. His medical history included hypertension under regular medication control, hepatitis B virus infection and hepatitis C virus infection. According to the patient's statement, he had a history of intravenous heroin addiction for more than 30 years.

**Fig. 1:**
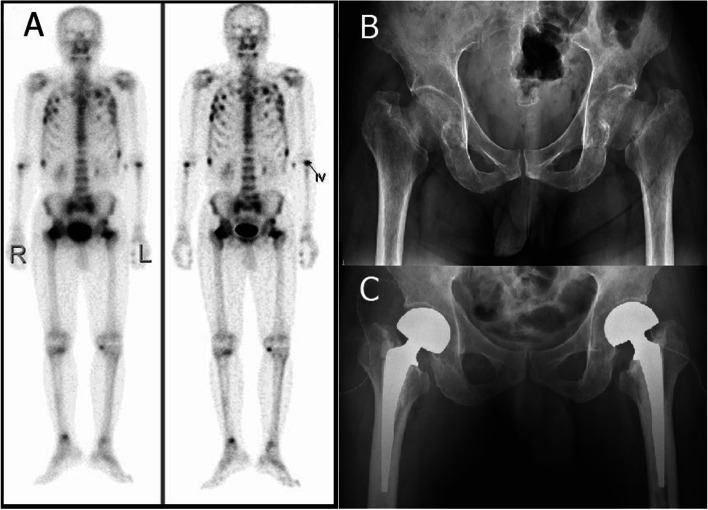
**A** Whole body bone scan: multiple hot spots of uptake located in the ribs, spine, sacrum ala, and bilateral hips. **B** Plain radiographs of the pelvis: bilateral femoral neck insufficiency fractures with displacement. **C** Post-operative plain radiographs of the pelvis

**Fig. 2:**
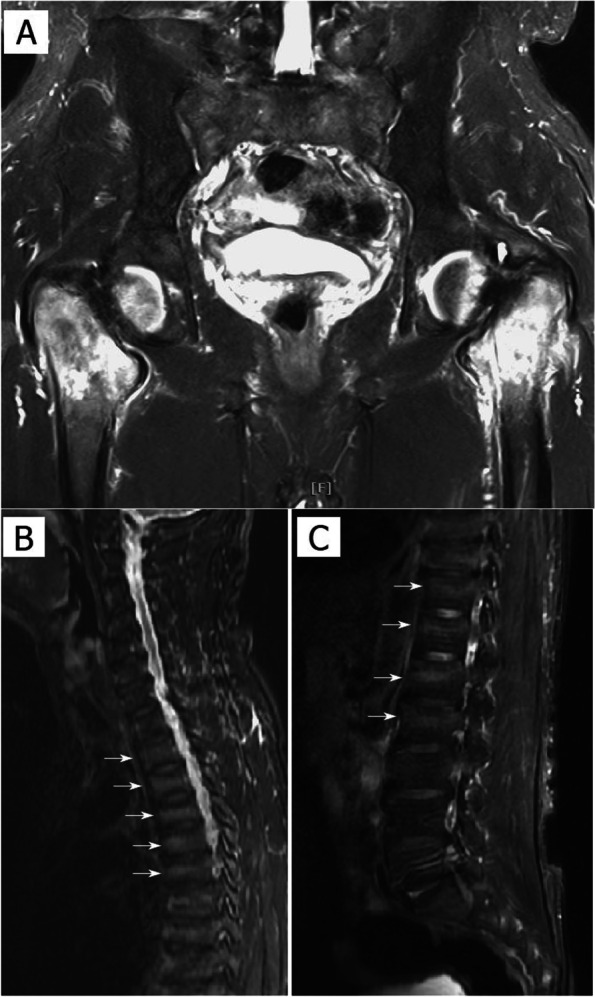
MRI of the pelvis and spine with STIR image. **A** High signal over the bilateral femoral neck; increased signal over the sacrum ala. **B** and **C** Multiple band-like lesions at the vert

Before he was referred to our orthopaedic department, he had gone to the medical department with complaints of decreased muscle power, general ache, and soreness over the bilateral lower limbs for approximately half a year. His bone mass index (BMI) was normal (22.8 kg/${m}^{2}$, reference range 18.5–24 kg/${m}^{2}$; body height 170 cm, body weight 65.9 kg). Plain radiographs of the spine revealed no remarkable findings. However, a whole-body bone scan showed multiple hot spots of uptake in the ribs, spine, sacroiliac joint and bilateral femoral neck (Fig. 1A). Further blood laboratory data revealed normal electrolyte values, including sodium (137 mmol/L, reference range 135–145 mmol/L) and potassium (3.9 mmol/L, reference range 3.5–5.0 mmol/L), albumin (3.6 g/dL, reference range 3.5–5.5 g/dL), intact parathyroid hormone (48.1 pg/ml, reference range 15–68.3 pg/ml), creatinine (0.85 mg/dL, reference range 0.7 ~ 1.2 mg/dl), tumour markers, including carcinoembryonic antigen(CEA), carbohydrate antigen 199(CA199), alpha fetoprotein(AFP), prostate-specific antigen(PSA) and monoclonal band values. However elevated alkaline phosphatase levels (365 U/L, reference range 34–104 U/L), and decreased inorganic phosphate (1.7 mg/dL, reference range 2.1–4.7 mg/dL), calcium (8.3 mg/dL, reference range 8.6–10.3 mg/dL) and 25-(OH)D3 (24.3 ng/ml, reference range: normal 30–100, insufficiency 20–30, scarcity < 20 ng/ml) levels were noted. Spot urine calcium/creatinine ratio showed normal (0.102, reference range < 0.2; urine calcium 12.4 mg/dL and urine creatinine 121.3 mg/dL). However, elevate spot urine phosphate was noted (54.7 mg/dL, reference range 3–18.75 mg/dL). Screening of fibroblast growth factor 23 revealed negative result. Bone marrow biopsy was performed and showed normocellular bone marrow. Magnetic resonance imaging (MRI) of the spine and hip demonstrated three important findings. First, there was an increased signal on the STIR image over the bilateral proximal femur with femoral neck insufficiency fractures. Second, there were insufficiency fractures at the bilateral sacral ala with increased signal on STIR images. Third, there were multiple band-like lesions, also known as looser zones, at the vertebrae of the thoracic and lumbar spines (Fig. 2).

While in the orthopaedic department, plain radiographs of the pelvis showed bilateral femoral neck insufficiency fractures with varus displacement (Fig. 1B). Surgical intervention was arranged. Further investigations into the causes of his osteoporosis were performed. Laboratory data showed normal thyroid function and osteocalcin levels and decreased testosterone levels (2.12 ng/ml, reference range 2.21–7.16 ng/ml). The screen for carbamazepine and phenobarbital was negative, and the urine screen for morphine was positive (> 1000 ng/ml, cut-off value 290 ng/ml). Bone densitometry was measured by DEXA (GE-Lunar/iDXA), and it revealed osteoporosis with a T score of minus 3.2 over spine and minus 4.0 over bilateral hips. Under the diagnosis of bilateral femoral neck insufficiency fracture caused by heroin-induced osteoporosis, bilateral hip hemiarthroplasty was performed (Fig. 1C), regular medication with vitamin D3 and calcium was prescribed, and detoxification treatment was arranged for him. Six months later, the level of 25-(OH)D3 recovered to 38.3 ng/ml, inorganic phosphate level recovered to 2.9 mg/dL, and calcium level recovered to 8.8 mg/dL. The patient could walk well with just one cane, and went back to light work after 3 months post-surgery.

## Discussion and conclusions

Previous studies have concluded that chronic use of opium and heroin both lead to several abnormal serum examination levels [5], and gonadal deficiency could be found in male with chronic heroin addiction [4], which is compatible with opioid-induced gonadal deficiency. Besides, heroin belongs to the opioid class of drugs, and their main effects are produced by binding with µ-opioid receptor in the brain. Therefore, heroin induced osteoporosis and opioid induced osteoporosis could be viewed as the same pathological process. Previous research mentioned the association between opium consumption and osteoporosis [6], and reduced bone density was observed in prolonged heroin addiction. Potential mechanisms of opioid-induced osteoporosis have been described in some studies [7–9]. Opioid addiction could influence bone mineral density and increase fracture risk by indirect and direct pathways. Opium addiction cause a reduction in the dietary intake of calcium, 25-(OH)D3 and phosphorus, which will result to hypocalcemia and hypophosphatemia. This could indirectly increase vulnerability to bone loss and osteoporosis. The direct pathway may involve the inhibition of osteoblastic tissue formation or gonadal deficiency [7]. A previous study stated that the function of osteoblasts is suppressed by opioid receptor binding [8, 9]. For gonadal deficiency, normally, the hypothalamus-pituitary–gonadal (HPG) axis is initiated by gonadotropin-releasing hormone (GnRH) secreted by the hypothalamus. Opioids block the release of GnRH through the µ-opioid receptor, and testosterone production is reduced accordingly [10]. Recently, the effect of testosterone on osteoblast activity has been elucidated [10, 11]. Testosterone could facilitate bone formation by increasing androgen receptor expression and regulating the expression of insulin-like growth factor-1 (IGF-1) and IGF-binding protein (IGF-BP) in osteoblasts. This would further suppress chondrocyte apoptosis and enhance differentiation [11]. Besides, Balodimos et al. mentioned that elevated bone turnover markers are higher in opioid-dependent patients, which is implicative of increased bone metabolism [12]. The 55-year-old male patient in our report had a history of heroin addiction for more than 30 years. He presented with bilateral femoral neck insufficiency fractures and severe osteoporosis with a T-score of minus 4.0 His laboratory data showed increased alkaline phosphatase, hypocalcaemia, hypophosphatemia, decreased testosterone levels, and lower level of 25-(OH)D3. The indirect pathway caused by hypocalcemia and hypophosphatemia and the direct effect resulted from gonadal deficiency and impaired osteoblast function because of reduced testosterone level attributed to the decreased release of GnRH by the opioid leading µ-opioid receptor binding collectively procured heroin-induced osteoporosis.

Although guidelines for monitoring and treating opioid-induced hypogonadism have not been proposed, several recommendations have been suggested [12–16]. Cessation of opioid use should be considered since the dose of morphine exceeding 100 mg per day could be related to the development of hypogonadism [15]. Moreover, testosterone levels were normalized in heroin-addicted patients undergoing opioid withdrawal [14]. In addition, testosterone replacement therapy for one year improved bone marrow density compared with the control group, but the difference was not significant [13]. Vitamin D stimulates intestinal calcium and phosphorus absorption, and calcium and phosphorous are indispensable elements for bone mineralization. Therefore, vitamin D and calcium supplementation for osteoporosis have shown an effective outcome [16]. In our case, discontinuation of opioid dose and adequate supplementation of calcium and vitamin D were prescribed. The level of calcium and 25-(OH)D3 recovered to normal level 6 months later.

A literature review described three case reports which revealed opioid-addicted patients with osteoporosis [17–19]. Lazarides et al. reported a case of a 27-year-old female with nine years of heroin addiction who presented with bilateral femoral neck fractures [17]. She had been amenorrhoeic for four years. Radiographic images, including MRI and plain film, revealed bilateral femoral neck stress fractures. Expect the DEXA scan showed a T-score of minus 2.7, there were not enough laboratory were mentioned in this case to support the evidence of heroin-induced osteoporosis. Hootkani et al. illustrated a case of 28-year-old male with eight months of heroin addiction who presented with bilateral femoral neck stress fractures [18]. Plain pelvic radiograph of the patient showed bilateral femoral neck stress fracture. Skeletal bone scan revealed no other abnormal findings except bilateral femoral head osteonecrosis and bilateral femoral neck fracture. However, there were not special laboratory tests to prove the opioid-induced osteoporosis. The DEXA was not available in this case. Gamache et al. reported a case of a 54-year-old female with thirty years of heroin addiction and menopause for more than twenty years [19]. In this case, multiple misshapen bones and pseudo-fractures were found that were attributable to severe osteomalacia. Hypocalcaemia, hypophosphatemia, and vitamin D deficiency were noted in this patient. However, the DEXA was also not available in this case. There is no specific guideline for treating opioid-induced hypogonadism. In our case, the crucial elements such as vitamin D, calcium and phosphorus are low, and the disease was caused by opioid. Cessation of opioid use and supplementation of vitamin D and calcium were recommended.

Nevertheless, the above cases only provide limited information of osteoporosis-related values. There were not enough laboratory data to support the direct and indirect pathway of heroin-addiction related osteoporosis. Further laboratory tests containing endocrine values, calcium, phosphorus, and 25-(OH)D3 could help to explain the relationship between heroin addiction and endocrine dysfunction, which further explicates the subsequent impaired bone density due to decreased testosterone or estrogen level. Other images, such as whole body bone scan and MRI, could help to detect the insufficiency fracture and exclude other systemic bone disease. In our case, we presented sufficient clinical laboratory data and radiology findings, which could support the diagnosis of opioid-induced osteoporosis presented with bilateral femoral neck insufficiency fractures.

The limitation of this study is short period of follow up. Although the level of calcium and 25-(OH)D3 recovered to normal level 6 months later, DEXA may need to be rechecked to assess the recovery of T score over a longer follow-up.

In conclusion, heroin-induced osteoporosis is not common clinically. However, early diagnosis and a better understanding of the mechanism underlying opioid-induced osteoporosis would improve the handling of this condition. This case report highlights the laboratory and radiology findings in an opioid-addicted patient and addresses the potential pathway by which osteoporosis is induced by opioids. When an unusual osteoporosis presents with insufficiency fractures, heroin-induced osteoporosis should be considered.

## Data Availability

The dataset(s) supporting the conclusions of this article is (are) included within the article.

## Abbreviations

BMI: Bone mass index
CEA: Carcinoembryonic antigen
CA 199: Carbohydrate antigen 199
AFP: Alpha fetoprotein
PSA: Prostate-specific antigen
MRI: Magnetic resonance imaging
HPG: Hypothalamus-pituitary–gonadal
GnRH: Gonadotropin-releasing hormone
IGF-1: Expression of insulin-like growth factor-1
IGF-BP: IGF-binding protein
